# A new 3-arylbenzofuran derivative EIE-2 reestablishes Treg-dependent tolerance in rheumatoid arthritis by targeting on Syk induced mTOR and PKCθ imbalance

**DOI:** 10.3389/fimmu.2025.1524491

**Published:** 2025-05-21

**Authors:** Ping Li, Xin Yin, Xinzhu Wen, Xuyu Li, Yuqing Wang, Hongyan Zhan, Hanqing Che, Chunsuo Yao, Qi Hou, Ziqian Zhang, Ruifang Zheng, Mingbao Lin

**Affiliations:** ^1^ Institute of Materia Medica, Chinese Academy of Medical Sciences and Peking Union Medical College, Beijing, China; ^2^ NHC Key Laboratory of Mental Health (Peking University), National Clinical Research Center for Mental Disorders (Peking University Sixth Hospital), Pharmacy Department, Peking University Sixth Hospital, Peking University Institute of Mental Health, Beijing, China; ^3^ Xinjiang Key Laboratory of Uygur Medical Research, Xinjiang Institute of Materia Medica, Urumqi, China

**Keywords:** EIE-2, rheumatoid arthritis, Treg-dependent tolerance, Syk, PKCθ/mTOR

## Abstract

**Introduction:**

Dysfunctional self-tolerance is thought to play a crucial role in the onset of rheumatoid arthritis (RA) pathogenesis. Poorly functioning regulatory T cells (Tregs) lead to extreme situations where self-tolerance is robustly disrupted. However, there are many uncertainties regarding its immunosuppressive pathways, especially concerning therapeutic drugs that are still in their infancy. Therefore, deciphering potential targets and developing novel drugs to ameliorate functional Tregs deficiency appears to be an efficient therapeutic approach for controlling RA.

**Methods:**

The therapeutic effects of EIE-2, a novel 3-arylbenzofuran derivative, were evaluated in collagen-induced arthritis (CIA) rats and carrageenan-induced paw edema mice *in vivo*, as well as in LPS-, PMA- or TNF-α-stimulated human CD4+ T cells (Jurkat), human synovial sarcoma cells (SW982) and primary isolated lymphocytes *in vitro*. The role of Syk in Treg-dependent tolerance and the mechanism of EIE-2 were explored using western blotting, quantitative reverse transcription PCR (qRT-PCR) and flow cytometry. Potential mechanistic targets were further validated through siRNA knockdown and molecular docking analysis.

**Results:**

EIE-2 significantly ameliorated arthritic symptoms and pathological damage in CIA rats by reducing pro-inflammatory cytokines and increasing anti-inflammatory factors in synovium and serum, and exhibited similar therapeutic effects in carrageenan-induced pedal edema mice. Moreover, EIE-2 potently suppressed the inflammatory responses in human synoviocyte SW982 cells, primary isolated lymphocytes and CD4+ Jurkat cells. Its therapeutic potential was associated with upregulation of Tregs during the active phase and downregulation during the inactive phase of RA. Mechanistically, EIE-2 modulated the PKCθ/mTOR ratio via Syk targeting, thereby restoring homeostasis in Tregs.

**Discussion:**

EIE-2 is a potential therapeutic candidate for RA. The underlying mechanism may involve its targeting on Syk to upregulate the PKCθ/mTOR ratio during the active phase of RA and downregulate the PKCθ/mTOR ratio during the inactive phase of RA, ultimately promoting Treg-dependent tolerance restoration.

## 1 Introduction

Rheumatoid arthritis (RA), a chronic autoimmune disease, is characterized by chronic abnormal synovial inflammation (synovitis) that attacks multiple joints, leading to cartilage damage, bone erosion and even disability as well as multiple systemic damage without proper treatment (1). Worldwide, RA affects approximately 0.5%-1% of all-age population with a prevalence two to three times higher in women than in men, placing a significant burden on the healthcare system (2, 3). Since the introduction of aspirin in the 1890s, glucocorticoids (GCs) in the 1940s, and methotrexate (MTX) in the 1980s for RA treatment, there have been substantial improvements in RA treatment drugs and their effects (4–6). Currently used disease-modifying anti-rheumatic drugs (DMARDs), including conventional synthetic DMARDs (csDMARDs), biological DMARDs (bDMARDs) and targeted synthetic DMARDs (tsDMARDs), have rapid effects and high efficiency which caninduce remission in many patients (7, 8). However, approximately 30% of patients do not achieve remission and no existing DMARDs can overcome immune disorders that play a vital role in the onset of RA pathogenesis (7). Furthermore, various adverse reactions are also inevitable with DMARDs (9). Therefore, novel targets and therapeutic drugs are necessary for RA treatment, especially for alleviating immune disorders and restoring immune balance in RA patients.

Regulatory T cells (Tregs), a CD4+ T-cell subtype characterized by CD25+ and FOXP3+ expression (10), are suggested to play a pivotal role of immune tolerance and homeostasis, suppressing autoimmune inflammatory events and maintaining peripheral tolerance (11). The dysfunction or impaired operation of Tregs is one of the mechanisms leading to severe compromise of self-tolerance during the progress of RA (12). In RA patients, significantly lower numbers of Tregs in the peripheral blood and a negative correlation with disease activity score 28 (DAS-28) have been reported (13, 14). Furthermore, although Tregs are enriched in the synovial fluid of RA patients, their inhibitory function is impaired due to the inflammatory milieu, resulting in an inability to inhibit the activation and maturation of immune cells (15–17). However, the numbers of Tregs are significantly increased in the peripheral blood of patients with inactive RA compared to active patients and healthy controls (18, 19), which increases risk of infection if they continue to use immunotherapeutic agents. Thus, restoring Tregs function to reestablish immune tolerance balance is promising as a specific intervention for RA.

But, the development, differentiation and activation of Tregs are complex, and how to overcome their impaired functions and regulate frequencies in RA is still unclear. The forkhead box P3 (FOXP3) is a key factor primarily involved in regulating the development and suppressive functions of Tregs (20, 21). The efficient functioning of Tregs requires not only FOXP3 but also other Treg-associated signatures and factors. Some studies have reported that spleen tyrosine kinase (Syk) plays a key role in RA progression (22, 23). Although Syk inhibitors have been used in clinical trials for the treatment of RA (24), differing results, along with the fact that the regulatory role of Syk in Tregs has not yet been fully elucidated, have limited their application. Mammalian target of rapamycin (mTOR) signaling has recently emerged as a key effector molecule in Syk activation (25) which seems to be particularly important for inhibiting FOXP3 transcription (26) and has been implicated in RA (27). Similarly, Syk activation affects protein kinase C (PKC)-activating signaling (28–32). PKCθ can enhance the expression of downstream transcription factors, such as activator protein 1 (AP-1) and nuclear factor of activated T cells (NFAT), thereby increasing FOXP3 expression (33–36). Additionally, evidence indicates that an inflammatory environment characterized by high levels of TNF-α is a principal etiological factor in the active phase of RA. It has been observed that TNF-α in the synovial tissue of RA patients inhibits FOXP3 phosphorylation, impedes the proliferation of Tregs, and reduces the secretion of the functional IL-10 and TGF-β (37, 38), which plays crucial roles in the activity and severity of RA (39). These issues prompted us to investigate the mechanisms behind selective modulation of the Syk/mTOR and Syk/PKCθ pathways in Tregs, and explore potential associations between the active inflammatory phase and TNF-α in RA.

Our previous research identified Amurensin H, a resveratrol dimer derived from Vitis amurensis Rupr, as a compound with substantial anti-inflammatory effects, exhibiting considerable efficacy in the treatment of asthma, COPD, and osteoarthritis (40–42). Furthermore, it functions as a competitive ATP inhibitor of Syk (43). Despite these benefits, Amurensin H is associated with certain toxic side effects and exhibits low bioavailability. To mitigate these drawbacks, we have undertaken a structural redesign, leading to the synthesis of EIE-2, a 3-arylbenzofuran derivative. This novel compound not only retains significant anti-inflammatory activity but also boasts reduced toxicity, addressing the limitations of its predecessor.

In this study, we demonstrated that EIE-2 has a potential to improve the effect on progression of collagen-induced arthritis and carrageenan-induced paw edema disease. We also found that EIE-2 targets Syk to modulate the ratio of PKCθ/mTOR, which in turn selectively affects Tregs level. In the model simulating the active phase of RA, EIE-2 increases the ratio to elevate Tregs levels, while it decreases the ratio to reduce Tregs level in the model simulating the inactive phase of RA. Ultimately, this restores Tregs homeostasis in RA animal models and alleviates the symptoms of RA while reducing the probability of infection. These results indicate that EIE-2 can become a potential lead compound for developing therapeutic agents against RA, and provides ideas for developing anti-RA drugs targeting Tregs homeostasis.

## 2 Materials and methods

### 2.1 Chemicals and animals

**Figure 1:**
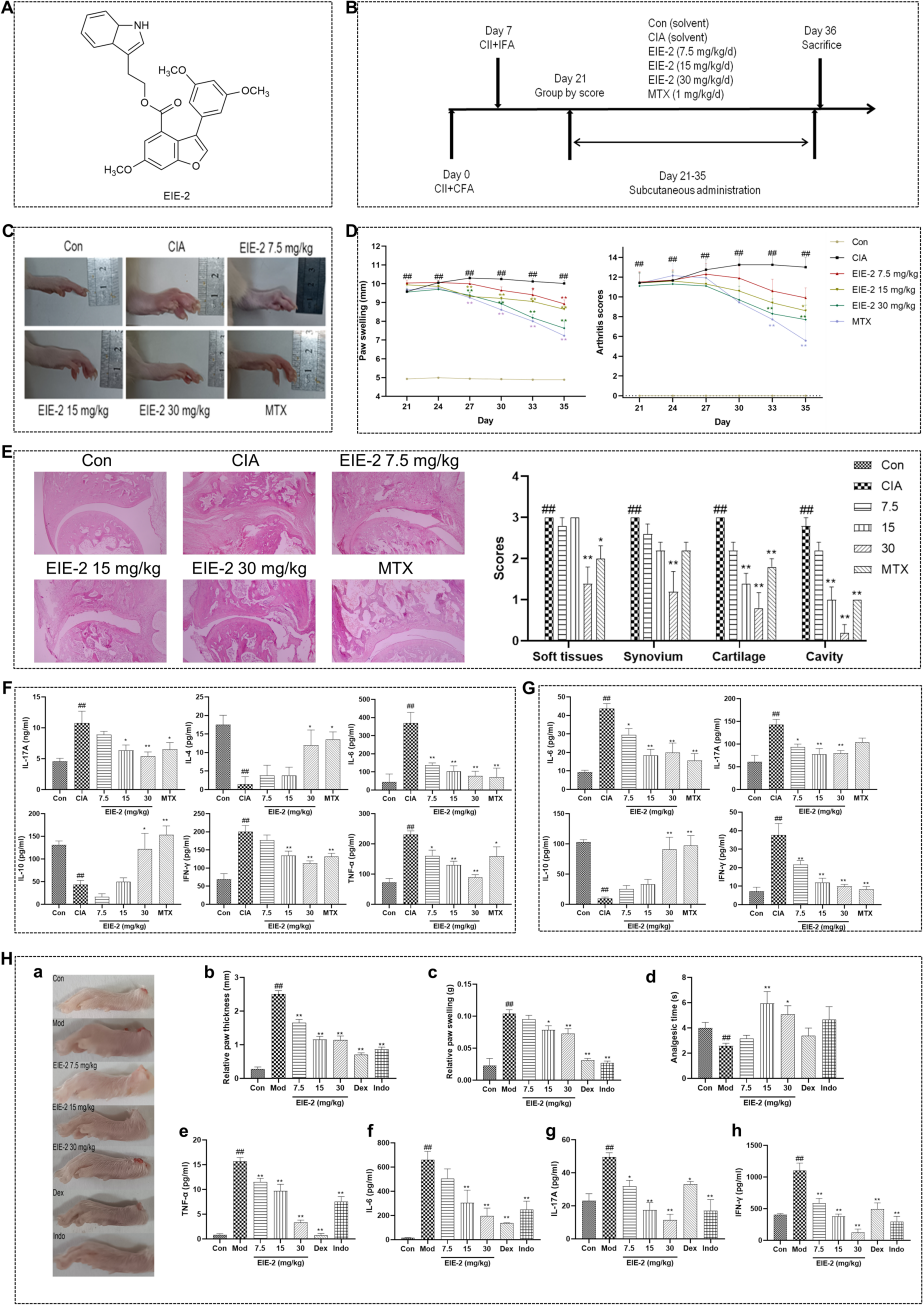
EIE-2 alleviates the progression of inflammation and joint injury in CIA rats and carrageenan-induced paw edema mice. **(A)** Chemical structure of EIE-2. **(B)** Timeline of the experimental protocol for CIA rats. **(C)** Photos of the paws of CIA rats were taken on day 35. **(D)** Paw swelling and arthritis scores were detected every three days beginning on day 21 (n=10-13). **(E)** The pathology results of the ankle joint with H&E staining (magnification 40×) (n=5). **(F, G)** The levels of IL-17A, IL-4, IL-6, IL-10, IFN-γ, and TNF-α in synovial homogenates **(F)** and of IL-6, IL-17A, IL-10, and IFN-γ in serum **(G)** were detected by ELISA (n=5). **(H)** The effects of EIE-2 on carrageenan-induced paw edema in mice. Paw photographs of carrageenan-induced paw edema mice (a), paw thickness at 2 h (b), paw swelling at 4 h (c) and analgesic time at 4 h (d) after carrageenan induction (n=10-12). (e-h) The levels of TNF-α, IL-6, IL-17A and IFN-γ in the serum were detected by ELISA (n=5). The data are shown as the mean ± SEM. ^#^p < 0.05 and ^##^p < 0.01 compared with the Con group, ^*^p < 0.05 and ^**^p < 0.01 compared with the CIA or Mod group.

EIE-2 (**Figure 1A**) was synthesized by Prof. Chunsuo Yao and identified by using ESI-MS and NMR. Methotrexate (MTX) was purchased from Solarbio (Beijing, China). Indomethacin (Indo) was purchased from Meilun (Dalian, China).

Male Wistar rats (160–180 g) were purchased from Vital River Laboratory Animal Technology Co., Ltd. (Beijing, China, SCXK (Jing) 2021-0011). Male Kunming mice (18–20 g) and male ICR mice (18–20 g) were purchased from Huafukang Biotechnology Co., Ltd. (Beijing, China, SCXK (Jing) 2019-0008). All animal experimental procedures were approved by the Animal Care & Welfare Committee of the Institute of Materia Medica, Chinese Academy of Medical Sciences & Peking Union Medical College (No. 00009281).

### 2.2 Collagen-induced arthritis in rats

The development timeline of CIA and treatment are shown in **Figure 1B**. Briefly (44), autoimmune arthritis was induced in Wistar rats by intradermally injecting 200 µg of bovine type II collagen (CII; Chondrex, WA, USA) emulsified with complete Freund’s adjuvant (CFA; Sigma-Aldrich, MO, USA) into the right caudal root on day 0, and 100 µg of CII emulsion into the left caudal root on day 7. According to the arthritis scores [0–4 scoring method (45)] and paw swelling (measured by using Vernier calipers), immunized rats were randomly divided into five groups (n=10-13) on day 21 and subcutaneously administered different doses of EIE-2 (7.5, 15, 30 mg/kg/d) or MTX (1 mg/kg/d) every day from day 21 to day 35. The control and model rats received the same volume of vehicle. After treatment, paw swelling and arthritis scores were measured every three days. On day 36, the rats were sacrificed, and the ankles, synovial knee joints, spleens, and serum were harvested for further analysis.

### 2.3 Carrageenan-induced paw edema in mice

Kunming mice were randomly divided into five groups (n=10-12) and treated with EIE-2 (7.5, 15, 30 mg/kg/d), dexamethasone (Dex 15 mg/kg/d), or indomethacin (Indo 15 mg/kg/d) for three days. The control and model mice were treated with the same volume of vehicle. On day 3, 50 µl of a 1% carrageenan solution was subcutaneously injected into the right paw of each mouse. Paw edema was assessed by measuring the difference in paw height (right paw minus left paw) at 2 hours post-injection and by determining the change in paw swelling (right paw weight minus left paw weight) at 4 hours post-injection. Afterward, the spleens and serum were collected for further analysis.

### 2.4 Histological analysis

The right ankle joints of CIA rats were fixed in 4% histocyte fixative, decalcified and embedded in paraffin. Hematoxylin and eosin (H&E) staining was used to evaluate joint severity using a pathology grading scale (inflammatory activity, synovial hyperplasia, cartilage degradation, and bone erosion) with four grades: 0 = normal, 1 = slight, 2 = moderate, and 3 = severe.

### 2.5 Cell culture and stimulation *in vitro*

Jurkat cells (a human CD4+ T lymphocyte cell line) and SW982 cells (a human synovial sarcoma cell line) were purchased from BeNa Culture Collection (BNCC) and cultured in RPMI 1640 medium supplemented with 10% fetal bovine serum (FBS), 100 U/ml penicillin and 100 µg/ml streptomycin in a 37°C incubator with 5% CO_2_.

SW982 cells in the logarithmic growth phase were seeded in 96-well plates (1x10^4^ per well) and incubated for 24 h. RPMI 1640 medium containing 2% FBS was replaced and then pre-protected with EIE-2 (2.5 μM, 5 μM, 10 μM), MTX (5 μM) for 2 h. Subsequently, the cells were stimulated for 24 h with 10 µg/ml LPS.

Spleen lymphocyte suspensions from ICR mice were prepared and resuspended in RPMI 1640 complete medium supplemented with 200 U/mL of mouse IL-2 (Peprotech, NJ, USA), 50 μM β-mercaptoethanol (Sigma-Aldrich, MO, USA), and 2 μg/mL of mouse anti-CD28 antibody (Biolegend, California, USA). The cells were then inoculated to 48-well plates that had been pre-coated with 5 μg/mL of mouse anti-CD3 antibody (Biolegend, California, USA) or coated with PBS. TNF-α (1 ng/mL) or EIE-2 (2.5, 5, and 10 μM) and MTX (5 μM) were added accordingly and treated for 72 h.

Jurkat cells in logarithmic growth phase were inoculated into 24 wells (1×10^6^ per well), pre-protected by adding EIE-2 (2.5, 5, 10 μM), MTX (5 μM) for 2h, and stimulated by adding PMA (100 ng/ml for ELISA or 20 ng/ml for FACS) or TNF-α(1ng/mL) for 24h.

For co-culture, Jurkat cells, pre-stimulated with PMA (100 ng/mL) for 24 h, were introduced into 24-well plates that had been pre-seeded with SW982 cells. The co-culture was then incubated for an additional 24 h, during which EIE-2 was added at varying concentrations of 2.5, 5 and 10 μM, along with MTX at a concentration of 5 μM.

For gene silencing, Jurkat cells were collected and seeded in RPMI 1640 medium supplemented with 2% FBS in 24-well plates for 1 h. siRNA-NC or siRNA-Syk (sequences: F: GCACUAUCGCAUCGACAAAdTdT, R: UUUUGUCGAUGCGAUAGUGCdTdT) was combined with High Performanc DNA/RNA Transfection Reagent (ZETA life, CA, USA) and introduced into the cells to achieve a final concentration of 100 nM. The mixture was then incubated for 4 h. Subsequently, EIE-2 (5 μM) was added to the culture and incubated for an additional 2 h. Finally, the cells were stimulated with either PMA (20 ng/mL) or TNF-α (1 ng/mL) and incubated for a total duration of 24 h.

All cell models do not remove the pre-administered drugs before stimulation. The culture supernatants were collected for further determination of cytokine levels, and the cells were subjected to qRT–PCR or flow cytometry analysis. MTS (Promega, WI, USA) was used for cell proliferation analysis.

### 2.6 Cytokine measurement

Enzyme-linked immunosorbent assay (ELISA) was used to determine inflammatory factor levels in *in vitro* cell culture supernatants and serum from experimental human, mouse or rat with ELISA kits for TNF-α, IFN-γ, IL-4, IL-8, IL-17A and IL-6 (Biolegend, San Diego, CA, United States); rat IL-10 (AssayGenie, Dublin, Ireland); and rat IL-4 and IFN-γ (Dayou, Shenzhen, China). All procedures were performed according to the manufacturer’s instructions, and the absorbance values at 450 nm and 570 nm were read by an enzyme marker (Power Wave XS2, BioTek, Virginia, USA).

### 2.7 Western blotting

Western blotting was performed according to standard methods. Briefly, protein extracted from tissues was separated by SDS–PAGE, transferred onto a PVDF membrane (Millipore, Bedford, MA, United States), the membrane was blocked using 5% (w/v) nonfat milk diluted in 1×TBST, and incubated with specific primary antibodies against p-Syk (Y525/526), Syk, GAPDH (Cell Signaling Technology, USA), p-mTOR, mTOR, FOXP3, p-PKCθ (S676), and PKCθ (Abcam, Cambridge, UK) at 4°C overnight and then incubated with horseradish peroxidase (HRP)-conjugated anti-rabbit secondary antibodies (Thermo Fisher, MA, United States) for 1 h. The bands were visualized and imaged by an enhanced chemiluminescence (ECL) imaging system (Clinx Science Instruments Co., Ltd., China). The relative intensities of the bands were analyzed by ImageJ.

### 2.8 Quantitative reverse transcription–polymerase chain reaction

Total RNA was extracted from cells using the TransZol Up Plus RNA Kit (TransGen, Beijing, China), and cDNA was obtained using the One-Step gDNA Removal reverse transcription kit (TransGen, Beijing, China). The commercial primers for human, mouse Syk, mTOR, PKCθ and GAPDH were synthesized by Huada Gene Technology Co., Ltd. (Primer sequences are shown in **Table 1**). The expression of the target gene was amplified using TransStart Top Green qPCR SuperMix (TransGen, Beijing, China) in a real-time PCR machine (MYGO PRO, IT-IS, Ireland). Gene expression was calibrated with the internal reference gene via the 2^-ΔΔCT^ method, and the relative target gene expression levels were obtained via control calibration (GAPDH).

**Table 1:** Primers used for qRT-PCR.

Gene name	Primer sequence (5′–3′)
H-GAPDH	F: TCATGACCACAGTCCATGCC R: AAGTGGTCGTTGAGGGCAAT
H-Syk	F: ATCGGCACACAGGGAAATGT R: ACTTTCTGTGGCCAGGCTTT
H-PKCθ	F: CGGAAGGAGATTGACCCACC R: TCAGTGCTCTGTCGGCAAAT
H-mTOR	F: CCATGGAACTCCGAGAGATGAG R: GGCAAATCTGCCAATTCGGG
M-GAPDH	F: ACCACAGTCCATGCCATCAC R: TCCACCACCCTGTTGCTGTA
M-Syk	F: CCTGATGTGGGAAGCGTTCT R: GGCCTGTTCTCCACATCGTAA
M-PKCθ	F: GGAGAGGCAGTGAACCCCTA R: TGTTCTTGCGGCATCTCTCC
M-mTOR	F: GCAGCTTTGAATTTGAAGGCCA R: ATGCCAAGACACAGTAGCGG

### 2.9 Flow cytometry analysis

The numbers of Tregs were detected by using flow cytometry analysis. Lymphocytes were obtained from the spleens of CIA rats, control mice or mice with paw edema by using a spleen lymphocyte separation solution (TBD, Tianjin, China). *In vivo* lymphocytes or *in vitro* cultured Jurkat Cell surfaces were stained at 4°C for 30 min using antibodies against CD4, CD25 to avoid light. For intracellular FOXP3 staining, cells were fixed and permeabilized using Foxp3/Transcription Factor Staining Buffer (eBioscience, CA, USA), and stained with FOXP3 antibody at room temperature 1 h. The flow antibodies, presented in **Table 2**, were used at the dilutions advised by the manufacturer’s guidelines. All stained cells were analyzed on the Flow Cytometer (2060R, ACEA NovoCyte, China).

**Table 2:** Antibodies used for flow cytometry.

Antibodies	Source	Clone number
Anti-human FOXP3 Alexa Fluor 647	Biolegend	259D
Anti-mouse/rat/human FOXP3 Alexa Fluor 647	Biolegend	150D
Anti-rat CD25 PE	Biolegend	OX-39
Anti-rat CD4 FITC	Biolegend	W3/25
Anti-mouse CD25 PE	Biolegend	PC61
Anti-mouse CD4 FITC	Biolegend	GK1.5

### 2.10 Wound healing assay

SW982 cells were seeded in a 6-well plate (1.5x10^6^ per well) with black line markers drawn in advance at the bottom and treated with EIE-2 (2.5, 5, 10 μM) and MTX (5 μM) for 24 h in RPMI 1640 medium supplemented with 2% FBS until they reached 85% confluence. Then, the cells were cultured in RPMI 1640 medium supplemented with 10% FBS after the lines were drawn, and free cells were removed by washing. Images were taken at the same position at 0 h and 24 h. Migration rate (%) = (0 h scratch area - 24 h scratch area)/0 h scratch area × 100%.

### 2.11 Molecular docking

The 3D structure of Syk (PDB ID: 6VOV) was subjected to heteroatom removal, cleaning, preparation and site definition in Discovery Studio software (BIOVIA, USA) for molecular docking. Syk (PDB ID: 6VOV) self-contained ligand GS-9876 (Lanraplenib) has been reported as a Syk inhibitor for the treatment of autoimmune diseases (46), using it as a positive control. EIE-2 was operated with a clean geometry, preparation and minimization before docking. Molecular docking of EIE-2 and GS-9876 at the active site of Syk (X: -13.586338, Y: 14.852337, Z: -4.834818, radius: 11.000000) was performed using the LibDock module in Discovery Studio software.

### 2.12 Statistical analysis

All experiments were conducted independently at least three times. Data was analyzed and plotted using GraphPad Prism 9 software. Data were presented as mean ± SEM. Statistical comparisons between two groups were made using Student’s t-test. Multiple group comparisons were performed using one-way analysis of variance (ANOVA) followed by Dunnett’s for multiple comparisons. P < 0.05 indicated statistical significance.

## 3 Results

### 3.1 EIE-2 alleviates the progression of inflammation and joint injury in CIA rats and carrageenan-induced paw edema mice

Collagen-induced arthritis (CIA) in rats is a common and widely used method to mimic pathological and functional injuries caused by rheumatoid arthritis in humans, CIA triggers chronic immunological synovial inflammation and cartilage damage in multiple joints after immunization with an emulsion of Freund’s adjuvant and type II collagen (47). Compared with those in the control group, the symptoms of arthritis in the CIA group dramatically progressed and maintained the highest intensity from day 21 to day 35. Significant increase in paw swelling and arthritis scores were detected in CIA rats, and these changes were obviously dose-dependently relieved by EIE-2 treatment (**Figures 1C, D**). Histological examinations also revealed significant synovitis and paw joint cartilage destruction in CIA rats, and EIE-2 treatment, as well as MTX treatment, significantly reduced this pathological injury (**Figure 1E**). Furthermore, EIE-2 dose-dependently decreased the levels of the inflammatory factors IL-17A, IL-6, IFN-γ and TNF-α and increased the levels of the anti-inflammatory factors IL-4 and IL-10 in synovial homogenates (**Figure 1F**) and serum (**Figure 1G**) of CIA rats.

To investigate the pharmacological effects of EIE-2 on acute joint inflammation, which leads to the occurrence of redness, swelling, pain and other symptoms at the affected site (48, 49), a carrageenan-induced paw edema mouse model was used. In comparison to the control group, mice treated with carrageenan exhibited pronounced paw edema (**Figures 1H**a–c), accompanied by a significant reduction in paw analgesic time (**Figure 1H**d). Furthermore, there was a marked elevation observed in the serum levels of inflammatory cytokines TNF-α, IL-6, IL-17A and IFN-γ (**Figures 1H**e–h). EIE-2 demonstrated a dose-dependent reduction in the aforementioned inflammatory response and associated symptoms.

Taken together, these *in vivo* data suggest that EIE-2 have significant anti-inflammatory effects that can slow the course of arthritis.

### 3.2 EIE-2 exerts an inhibitory effect on the inflammatory responses within the SW982, murine splenic lymphocytes, Jurkat cells, as well as in a co-culture system comprising SW982 and Jurkat cells *in vitro*

In RA, immune cells that infiltrate the synovium collaborate with structural cells to create an inflammatory microenvironment. This collaboration results in altered cell behaviors that fuel inflammation, promote tissue damage, and hinder the resolution of inflammation (50).

**Figure 2:**
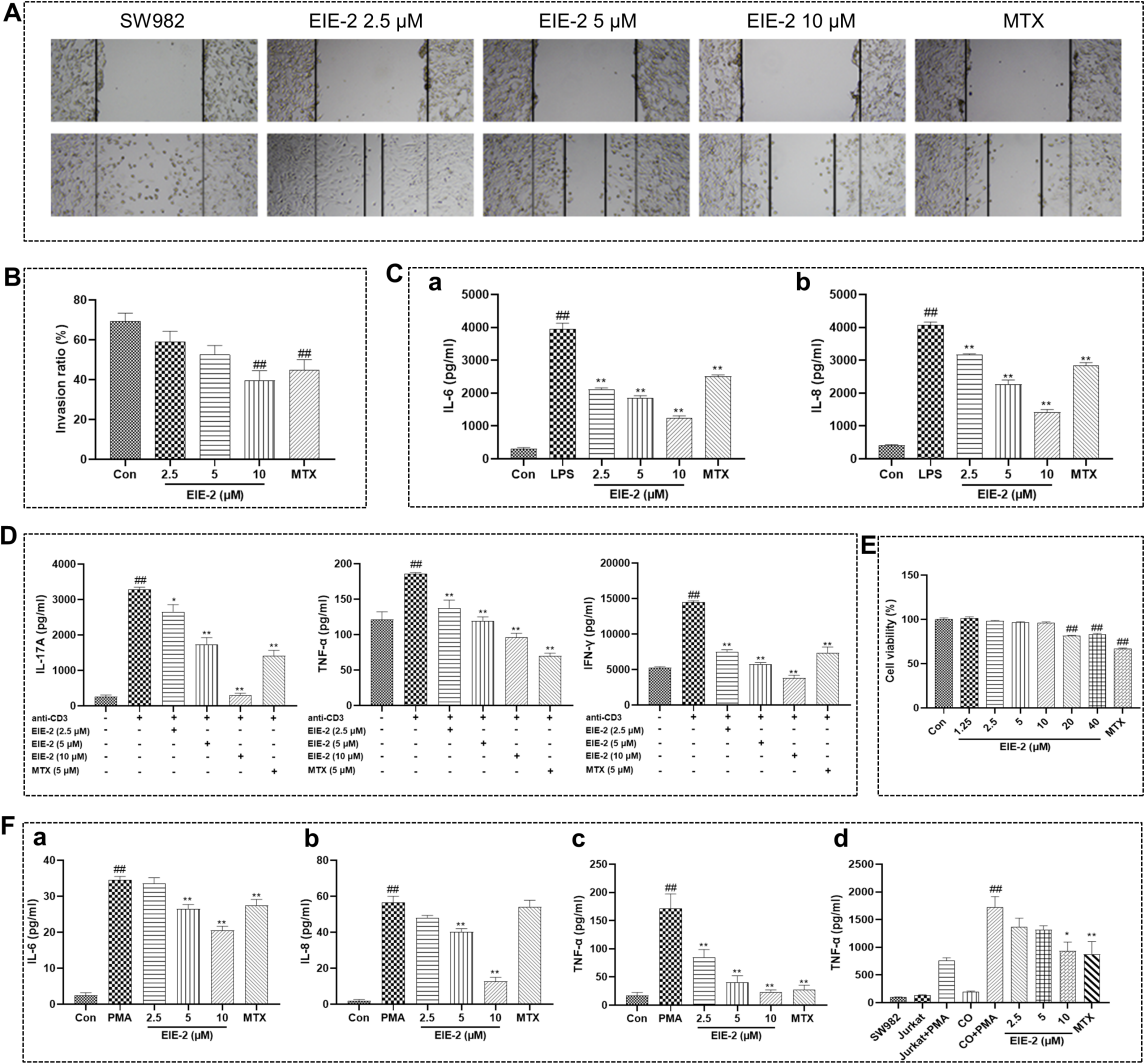
EIE-2 exerts an inhibitory effect on the inflammatory responses within the SW982, murine splenic lymphocytes, Jurkat cells, as well as in a co-culture system comprising SW982 and Jurkat cells *in vitro*. **(A)** Wound areas were photographed by an inverted research microscope (magnification ×40), **(B)** Wound areas were analyzed by ImageJ (n=15). **(C)** The levels of secreted IL-6 (a) and IL-8 (b) in LPS-treated SW982 cells were detected by ELISA (n=5). **(D)** The levels of inflammatory factor production in activated primary spleen lymphocytes from ICR mice (n=5). **(E)**The proliferation of Jurkat cells treated with EIE-2 was measured by MTS (n=6). **(F)** The levels of IL-6 (a), IL-8 (b) and TNF-α (c) secretion in PMA-treated Jurkat cells and TNF-α secretion in co-cultured cells (d) were detected by ELISA (n=5). The data are shown as the mean ± SEM. ^#^p < 0.05 and ^##^p < 0.01 compared with the Con group, ^*^p < 0.05 and ^**^p < 0.01 compared with the LPS or PMA group.

Fibroblast-like synoviocytes (FLS) represent a structural cells within synovial tissue, and their dysregulated migration and inflammatory responses are pivotal in the pathogenesis of RA (51, 52). Moreover, activated FLS demonstrated the capacity to express receptor activator of nuclear factor-κB (NF-κB) ligand (RANKL), which in turn triggered osteoclast development and functional maturation. This process not only promoted bone destruction but also induced the expression of matrix metalloproteinases (MMPs), thereby expediting the degradation of cartilage (53).The human synovial cell line, SW982, is a useful tool for studying the expression of inflammatory cytokines in FLS (54–56). The results show that treatment with EIE-2 dose-dependently inhibited SW982 cell migration (**Figures 2A, B**) and reduced the secretion of the inflammatory factors IL-6 and IL-8 as effectively as MTX treatment (**Figure 2C**).

Lymphocytes are among the immune cells that infiltrate the synovial tissue, and their activation is intricately linked to the inflammatory response characteristic of RA. We found that EIE-2 treatment significantly suppressed the inflammatory activation of mouse primary spleen lymphocytes *ex vivo* by inhibiting, IL-17A, TNF-α and IFN-γ secretion (**Figure 2D**).

Lymphocytes in the synovial membrane are mostly CD4+ T cells that release pro-inflammatory cytokines and chemokines. These substances help destroy cartilage and bone in joints (57). To further investigate the *in vitro* anti- inflammatory effects of EIE-2, a CD4+ Jurkat cell line was used. The results indicate that EIE-2 significantly inhibited the secretion of IL-6, IL-8, and TNF-α in PMA-treated Jurkat cells (**Figures 2F**a–c) without cytotoxicity (**Figure 2E**).

To replicate the inflammatory microenvironment of the synovial cavity in an *in vitro* setting, we established a co-culture system consisting of Jurkat cells and SW982 cells. Our results showed that 10 μM EIE-2 significantly inhibited TNF-α levels in the microenvironment of PMA-induced synovial inflammation (**Figure 2F**d).

Collectively, the outcomes of *in vitro* experiments provide further evidence that EIE-2 effectively suppresses the migration and inflammatory reactions of synoviocytes, as well as the inflammatory responses of lymphocytes, particularly CD4+ T cells. Additionally, EIE-2 was observed to mitigate the inflammatory response within the simulated synovial lumen microenvironment, which was created through the co-culture of synoviocytes and CD4+ T cells.

### 3.3 EIE-2 increased the proportion of Tregs in the model simulating the active phase of RA while decreased their proportion in the model simulating the inactive phase

**Figure 3:**
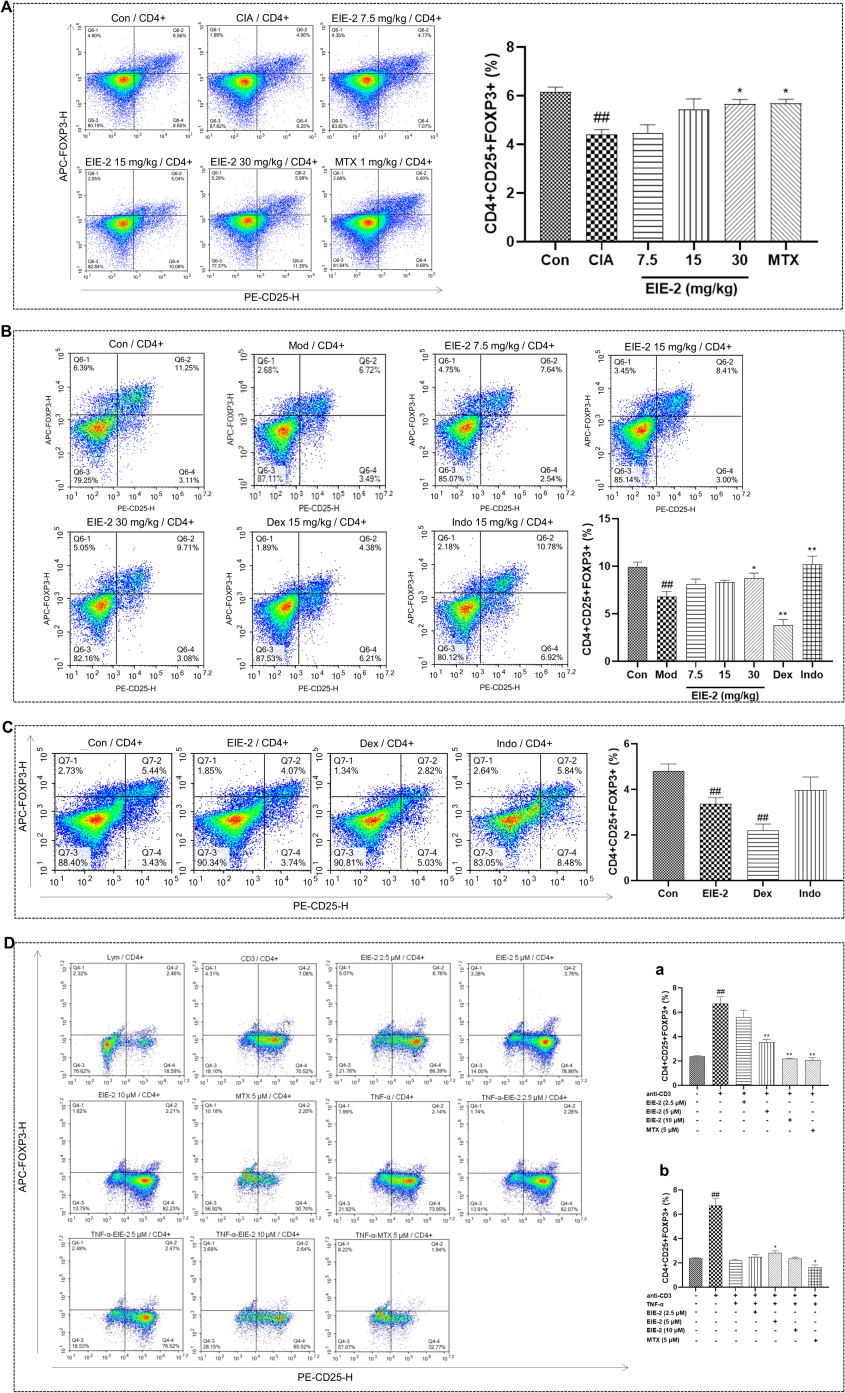
EIE-2 increased the proportion of Tregs in the model simulating the active phase of RA while decreased their proportion in the model simulating the inactive phase. FACS analysis was employed to assess the regulatory influence of EIE-2 on the CD4+CD25+FOXP3+ Tregs population. Percentage of Tregs in splenic lymphocytes from CIA model rats **(A)**, carrageenan-induced paw edema model mice **(B)**, and control mice **(C)**. Percentage of Tregs in normal (**D**, a) and TNF-α-induced (**D**, b) isolated primary splenic lymphocytes. The data are shown as the mean ± SEM (n=5). ^#^p < 0.05 and ^##^p < 0.01 compared with the Con group, ^*^p < 0.05 and ^**^p < 0.01 compared with the CIA or Mod group.

Patients with RA exhibit dysfunctional Tregs that are compromised in their anti-inflammatory and immunosuppressive capabilities. It has been reported that the level of Tregs is lowest in patients with active RA but highest in patients with inactive RA (19). Here, by using FACS, the effects of EIE-2 on Tregs regulation were detected. The findings revealed a marked reduction in the Tregs population in CIA model rats (**Figure 3A**) and carrageenan-induced paw edema model mice (**Figure 3B**), aligning with the features of the active phase of RA. Notably, EIE-2 administration significantly reversed this decline. Meanwhile, EIE-2 treatment significantly reduced the proportion of Tregs in control mice (**Figure 3C**), suggesting that EIE-2 has the potential to alleviate the elevated proportion of Tregs caused by the inactive phase of RA.

It has been reported that TNF-α suppresses the activity of Tregs by downregulating the expression of FOXP3 in RA (58). Moreover, therapy with anti-TNF antibodies facilitates the proliferation of Tregs and augments their inhibitory functionality (59–61). Therefore, we evaluated the regulation of Tregs by EIE-2 *in vitro* by constructing a model of TNF-α-induced decrease in Tregs of isolated splenic primary lymphocytes and a model of increase in Tregs of normal isolated splenic primary lymphocytes. Consistent with the *in vivo* results, EIE-2 decreased the percentage of CD4+CD25+FOXP3+ Tregs in normal isolated splenic primary lymphocytes (**Figure 3D**a), meanwhile, EIE-2 treatment with 5µM significantly reversed the TNF-α-induced reduction ratio of CD4+CD25+FOXP3+ Tregs (**Figure 3D**b).

These results implied that EIE-2 has a bidirectional role in regulating Tregs, as demonstrated by its ability to both elevate the proportion of Tregs in the model simulating the active phase of RA and inhibit the abnormal rise in the proportion of Tregs in the model simulating the inactive phase of RA.

### 3.4 The Syk/PKCθ/mTOR signaling pathway is subject to bi-directional control by EIE-2 across the models simulating the active and inactive phases of RA

**Figure 4:**
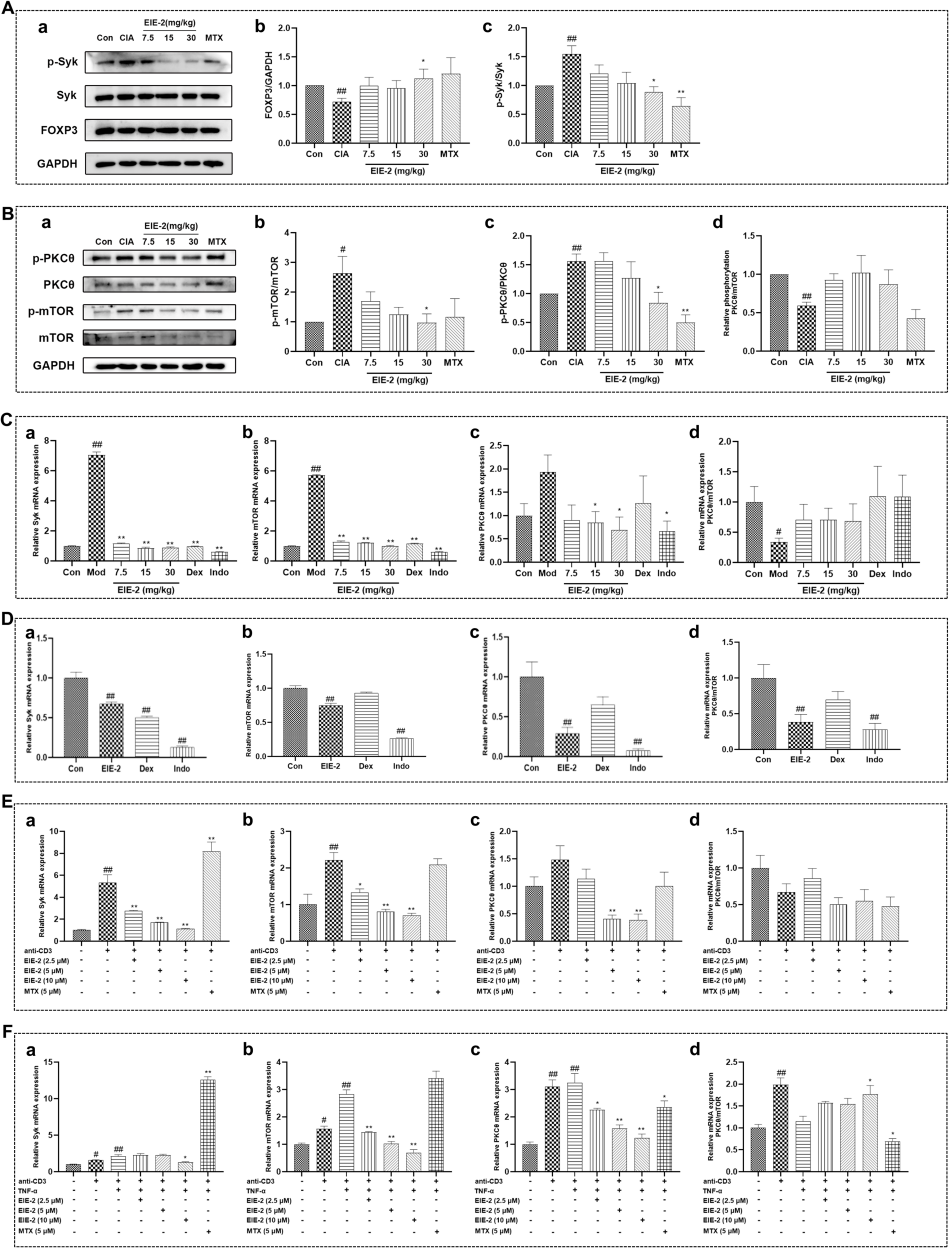
The Syk/PKCθ/mTOR signaling pathway is subject to bi-directional control by EIE-2 across the models simulating the active and inactive phases of RA. (**A**, a) Representative western blot images of p-Syk, Syk or FOXP3 in spleen of CIA rats. (**A**, b, c) Relative expression of FOXP3 to GAPDH, p-Syk to Syk. (**B**, a) Representative western blot images of p-PKCθ, PKCθ, p-mTOR or mTOR in spleen of CIA rats. (**B**, b–d) Relative expression of p-mTOR to mTOR, p-PKCθ to PKCθ and p-PKCθ/PKCθ to p-mTOR/mTOR (n=5-6). The transcription levels of Syk(a), mTOR(b), PKCθ (c) and the mRNA ratio of PKCθ to mTOR(d) in the spleen of mice with carrageenan induced paw edema **(C)** (n=3-6), in the spleen of control mice **(D)** (n=7), in isolated primary splenic lymphocytes without TNF-α **(E)** (n=3-6) and in TNF-α-induced isolated primary splenic lymphocytes **(F)** (n=3) were detected by qRT-PCR. The data are shown as the mean ± SEM. ^#^p < 0.05 and ^##^p < 0.01 compared with the Con group, ^*^p < 0.05 and ^**^p < 0.01 compared with the CIA or Mod group.

To clarify the potential mechanism by which EIE-2 regulates Tregs, we investigated signaling pathways related to RA and Tregs using models corresponding to the active phase of RA (CIA rats, carrageenan-induced paw swelling mice, and TNF-α-induced in isolated primary spleen lymphocyte Tregs reduction model), as well as models corresponding to the inactive phase of RA (control mice and in isolated primary spleen lymphocyte Treg increase model). It has been reported that Syk plays a role in modulating the pathological progression of RA (62, 63). Additionally, both PKCθ and mTOR are implicated in the regulation of Tregs function. Notably, PKCθ is involved in Tregs differentiation *in vivo (* 64), whereas the activation of mTOR has been observed to have an inhibitory effect on Tregs (26, 65). The findings revealed that EIE-2 significantly reduced the phosphorylation levels of Syk, mTOR, and PKCθ proteins in the spleens of CIA rats in a dose-dependent manner. This treatment led to an overall increase in the PKCθ/mTOR ratio at protein phosphorylation levels, accompanied by a notable upregulation of FOXP3 protein expression (**Figures 4A, B**). The same gene transcription results were observed in the spleens of mice with carrageenan-induced paw edema treated with EIE-2 (**Figure 4C**). In the spleens of control mice, EIE-2 treatment was observed to significantly lower the mRNA expression levels of Syk, PKCθ, and mTOR. However, this intervention resulted in an overall reduction in the ratio of the PKCθ/mTOR transcription levels (**Figure 4D**).

Furthermore, *in vitro* results revealed that EIE-2 dose-dependently suppressed the transcription of Syk, PKCθ, and mTOR in normal primary spleen lymphocytes, leading to an overall decline in the ratio of PKCθ/mTOR transcription level (**Figure 4E**), consequently, this led to a decrease in the proportion of Tregs. Contrarily, in isolated primary spleen lymphocytes stimulated with TNF-α, EIE-2 treatment was observed to elevate the PKCθ/mTOR ratio at the transcription level (**Figure 4F**), which corresponded to an enhanced Tregs ratio. These findings are in alignment with the *in vivo* observations.

In summary, EIE-2 effectively inhibits the protein phosphorylation levels of Syk/PKCθ/mTOR *in vivo* in animal models, and also effectively suppresses the gene transcription levels of Syk/PKCθ/mTOR *in vitro* in cell models. Notably, EIE-2 exhibits a unique bi-directional regulatory effect on the PKCθ/mTOR ratio, up-regulating it in the model simulating the active phase of RA and down-regulating it in the model simulating the inactive phase.

### 3.5 The influence of EIE-2 on FOXP3 expression in Tregs was positively associated with its capacity to regulate the PKCθ/mTOR ratio

**Figure 5:**
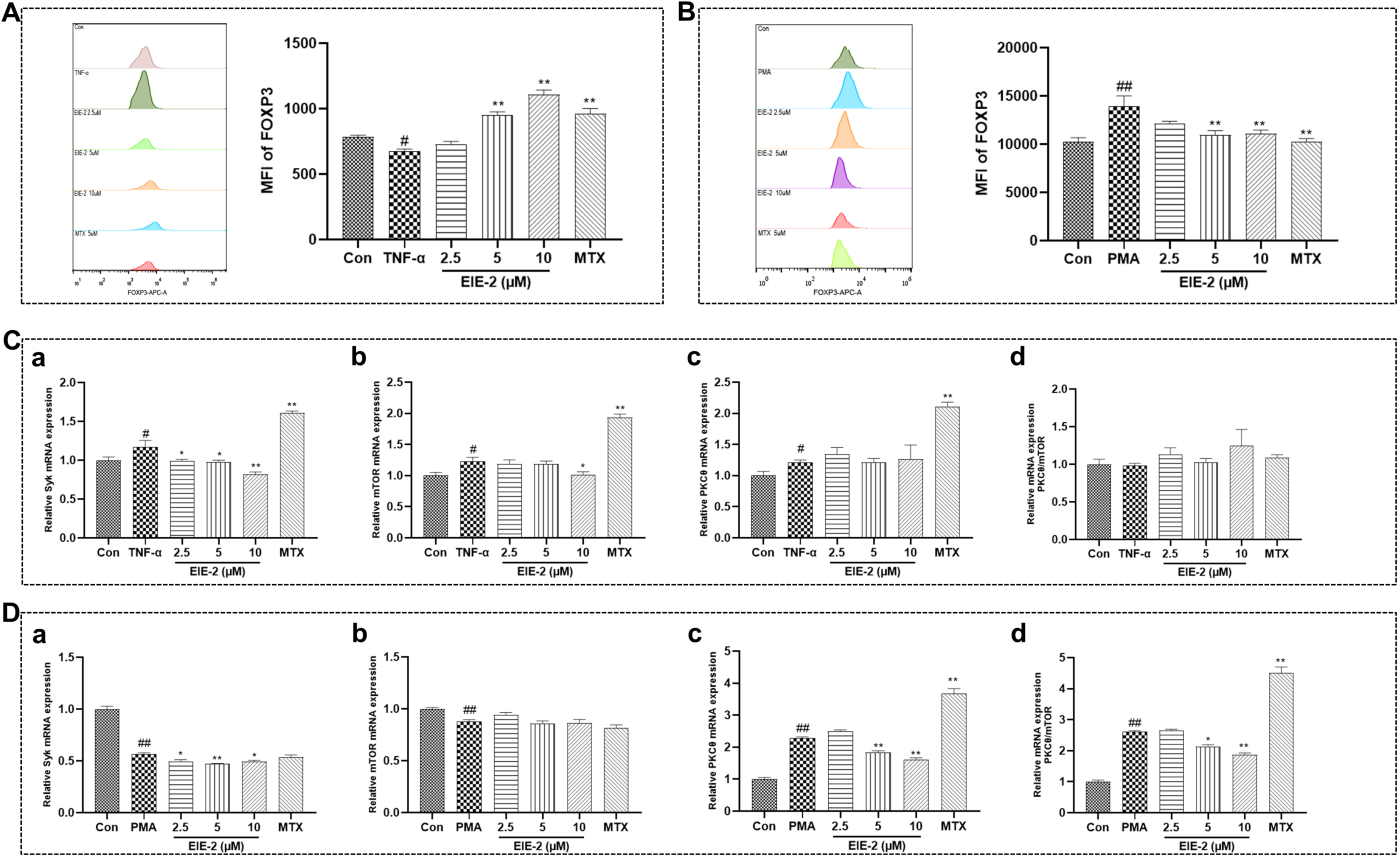
The influence of EIE-2 on FOXP3 expression in Tregs was positively associated with its capacity to regulate the PKCθ/mTOR ratio. FACS was used to detect the effect of EIE-2 on FOXP3 expression in Jurkat cells treated with TNF-α **(A)** and PMA **(B)** (n=5). The effect of EIE-2 on the transcription levels of Syk (a), mTOR (b), PKCθ (c) and the mRNA ratio of PKCθ to mTOR (d) in TNF-α **(C)** (n=4-6) and PMA **(D)** (n=3-8) treated Jurkat cells was detected by qRT-PCR. The data are shown as the mean ± SEM. ^#^p < 0.05 and ^##^p < 0.01 compared with the Con group, ^*^p < 0.05 and ^**^p < 0.01 compared with the PMA or TNF-α group.

To explore the regulatory mechanisms of Tregs, we extended our investigations using the CD4+ T cell line Jurkat. Firstly, we simulated the reduction in Treg numbers observed in patients with active RA by inducing a decrease in FOXP3 expression in Jurkat cells via treatment with TNF-α. Our findings demonstrated that EIE-2 treatment significantly augmented FOXP3 expression (**Figure 5A**). This effect was associated with a decrease in the mRNA levels of Syk and mTOR, while the mRNA levels of PKCθ remained unchanged, resulting in an increase in the PKCθ/mTOR ratio at the transcription level, albeit the change was not statistically significant (**Figure 5C**).

Subsequently, to mimic the increase in Treg numbers seen in patients with inactive RA, we induced the upregulation of FOXP3 expression in Jurkat cells using PMA. The data revealed that EIE-2 treatment significantly attenuated FOXP3 expression (**Figure 5B**). This reduction was associated with decreased mRNA levels of Syk and PKCθ, while the mRNA levels of mTOR were unaffected, leading to a decrease in the PKCθ/mTOR ratio at the transcription level (**Figure 5D**).

Overall, the preferential suppression of PKCθ or mTOR, and consequently the alteration of the PKCθ/mTOR ratio through Syk inhibition within a TNF-α-perturbed microenvironment, emerges as a pivotal molecular tactic by which EIE-2 facilitates the reestablishment of Treg homeostasis.

### 3.6 EIE-2 bi-directionally modulates of the PKCθ/mTOR ratio in Tregs across the models simulating the active and inactive phases of RA through direct targeting on Syk

**Figure 6:**
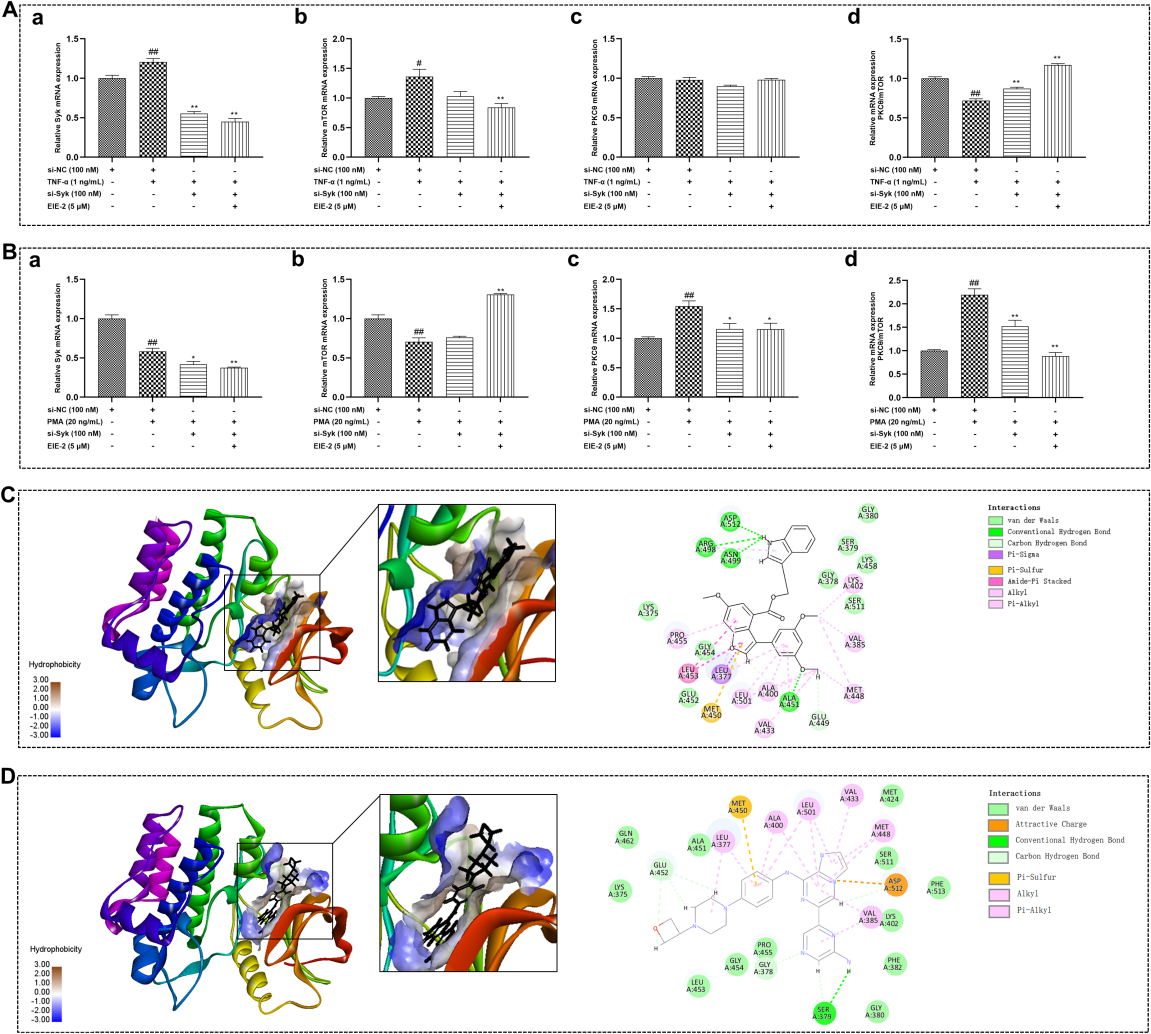
EIE-2 bi-directionally modulates of the PKCθ/mTOR ratio in Tregs across the models simulating the active and inactive phases of RA through direct targeting on Syk. The effects of siRNA-Syk and EIE-2 on the transcription levels of Syk (a), mTOR (b), PKCθ (c) and the mRNA ratio of PKCθ to mTOR (d) in Jurkat cells treated with TNF-α **(A)** and PMA **(B)** were detected by qRT-PCR. Molecular docking studies of EIE-2 **(C)** and GS-9876 **(D)** into the active sites of Syk. The data are shown as the mean ± SEM (n=3-6). ^#^p < 0.05 and ^##^p < 0.01 compared with the control group (transfection siRNA-NC), ^*^p < 0.05 and ^**^p < 0.01 compared with the PMA or TNF-α group.

To rigorously validate that EIE-2 targets Syk to bi-directionally regulate the PKCθ/mTOR ratio in Tregs in the models simulating different active phases of RA, we employed siRNA targeting Syk. Transfection of siRNA-NC did not alter the effects of TNF-α and PMA on gene expression in jurkat cells. Transfection with siRNA-Syk in TNF-α-treated Jurkat cells led to decreased transcript levels of Syk and mTOR, with no significant impact on PKCθ expression, thereby reversing the TNF-α-mediated decline in the PKCθ/mTOR ratio at the transcription level. This effect was potentiated in the presence of EIE-2 (**Figure 6A**). In PMA-treated Jurkat cells, Syk knockdown resulted in reduced transcript levels of Syk and PKCθ, while mTOR expression remained largely stable, counteracting the PMA-induced increase in the PKCθ/mTOR ratio at the transcription level, an effect that was similarly enhanced by EIE-2 (**Figure 6B**). EIE-2 is proposed to function as an inhibitor of Syk, demonstrating an inhibition profile akin to that of Syk-specific siRNA. The concurrent application of EIE-2 and Syk-specific siRNA synergistically amplified the inhibition of Syk expression in Jurkat cells, thereby eliciting a more marked regulatory impact on subsequent signaling cascades.

Furthermore, docking simulations of EIE-2 (**Figure 6C**) and the Syk inhibitor GS-9876 (**Figure 6D**) with Syk were performed to clarify whether EIE-2 directly interacts with Syk. Human Syk (PDB ID: 6VOV) docked with EIE-2 at the active site with a Libdock score of 126.104 and with GS-9876 with a Libdock score of 122.059. In the 2D docking interaction diagram (**Figure 6C**), EIE-2 formed conventional hydrogen bonds with amino acid residues ASP512, ARG498, ASN499, ALA451, and carbon-hydrogen bonds with GLU449, and the rest of the van der Waals, Pi-sigma, and Pi-Sulfur, Amide-Pi Stacked, Alkyl and Pi-Alkyl interactions are also present. The findings imply that EIE-2 possesses favorable binding characteristics to Syk.

Taken together, these data further indicated that EIE-2, by directly targeting Syk, resulted in an upregulation of the PKCθ/mTOR ratio at the transcription level in Jurkat cells under conditions that mimic the active phase of RA, as induced by TNF-α. Conversely, during the inactive phase of RA, as simulated by PMA, EIE-2 is associated with a downregulation of the PKCθ/mTOR ratio at the transcription level in Jurkat cells.

## 4 Discussion

**Figure 7:**
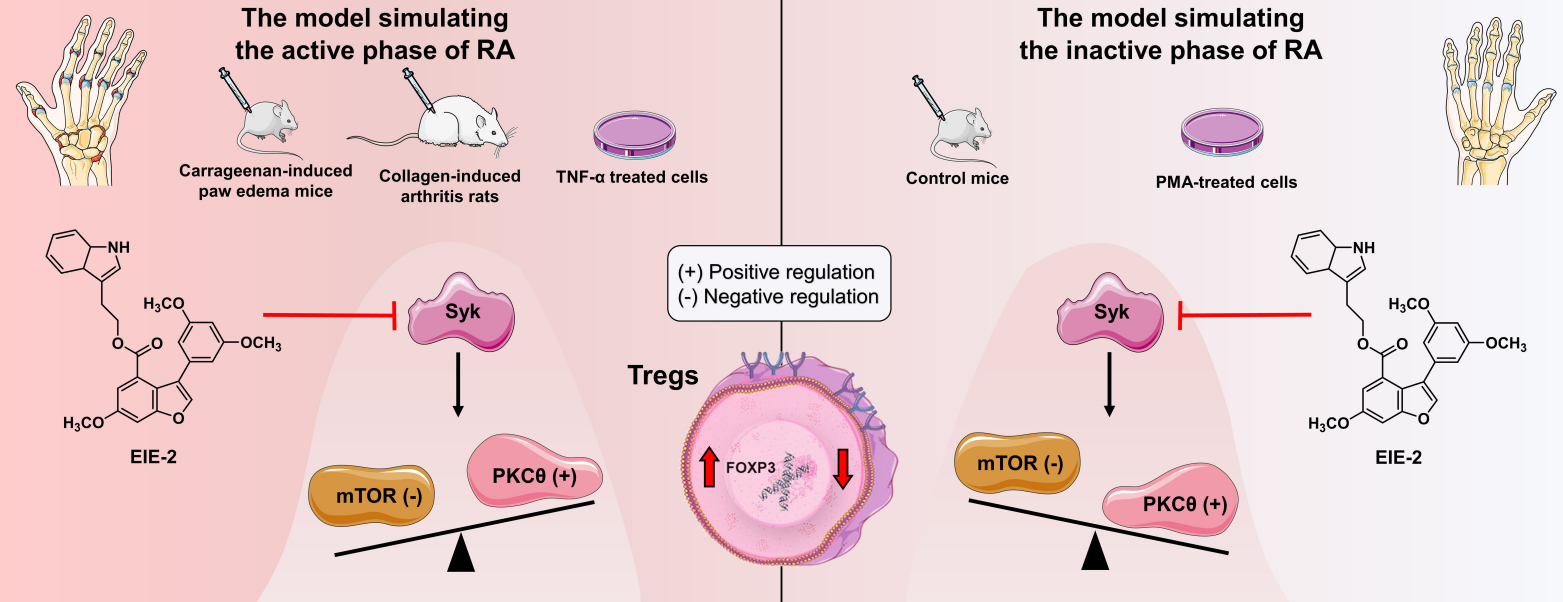
Summary of the potential molecular mechanisms for EIE-2 mitigation of RA Symptoms. EIE-2 inhibits Syk, thereby upregulating the PKCθ/mTOR ratio in the model simulating the active phase of RA to increase the number of Tregs, and downregulating the PKCθ/mTOR ratio in the model simulating the inactive phase of RA to reduce the Treg population. Black arrows and balance depict the possible pathological molecular mechanisms of RA, while red arrows illustrate the therapeutic intervention of EIE-2.

Rheumatoid arthritis (RA) is characterized as a chronic autoimmune disease that leads to disability and various types of systemic damage but is not adequately treated. The progression of RA is associated with abnormal synovial invasion, effector T-cell activation, impaired immune tolerance dependent on Tregs, and cytokine release (12, 52, 66–68). Among these, immune disorders caused by impairment in Treg-dependent immune tolerance play a vital role in the onset of RA pathogenesis (7). However, no existing DMARDs can effectively address these immune disorders (7). Therefore, it is important to identify candidates that can alleviate immune disorders and restore the immune balance of Tregs in patients with RA. Several drugs that affect Tregs numbers or function have shown efficacy in the treatment of RA (69–71). In the present study, we have demonstrated that EIE-2, A new 3-arylbenzofuran derivative, exhibits remarkable efficacy in mitigating inflammation and joint damage in both CIA rats and carrageenan-induced paw edema mice. Furthermore, our *in vitro* studies have shown that EIE-2 exhibits significant anti-inflammatory properties and the capability to inhibit synovial cell migration. The therapeutic effects of EIE-2 are realized by inhibiting Syk and thus modulating the ratio of PKCθ/mTOR, which ultimately promotes Treg-dependent tolerance restoration (**Figure 7**). Thus, EIE-2 is expected to be a therapeutic candidate for RA.

Tregs, a group of CD4+ immunosuppressive cells, maintain immune homeostasis and suppress the overactivated autoimmune response to reduce the severity of rheumatoid arthritis (72). Concurrently, Tregs play a protective role in bone homeostasis by inducing apoptosis of osteoclast precursors and suppressing the production of osteoclasts, thereby mitigating bone resorption (73). Clinical studies reported that the number and inhibitory activity of Tregs are decreased in active RA patients but increased in inactive RA patients (18, 74), which is mainly associated with TNF-α, a major pathogenic inflammatory factor that plays crucial roles in the activity and severity of RA (37–39, 58). In the current study, a significant reduction in the number of Tregs was observed in the spleens of CIA rats and carrageenan-induced paw edema mice, as well as in TNF-α-treated primary splenic lymphocytes. This finding indicates that the animal and cellular models employed in this research effectively replicate the clinical characteristics of patients with active RA. Prior research has indicated that the administration of DMARDs is linked to an elevated risk of opportunistic infections (75). Furthermore, the immunosuppressive properties of bDMARDs further heighten the infection risk in patients (76). Interestingly, EIE-2 significantly increased the number of Tregs not only in the RA active phase model, but also significantly reduced the number of Tregs in models representing the inactive phase of RA, such as spleens of control mice and untreated primary splenic lymphocytes. These results suggest that restoring the balance of Treg tolerance may be a major mechanism by which EIE-2 alleviates the pathological progression of RA. Notably, the protective effects of EIE-2 against cartilage damage in CIA rats can be ascribed to its dual actions: the inhibition of FLS migration and inflammatory response, and the restoration of Treg functions, which include the suppression of osteoclastogenesis and the induction of osteoclast apoptosis. Should EIE-2 be combined with DMARDs, it holds the promise of not only enhancing the effectiveness of RA treatment but also capitalizing on EIE-2’s immunomodulatory potential to counteract the adverse effects and risks associated with prolonged DMARDs therapy. Such attempts have been reported extensively in previous studies (77).

However, the mechanisms by which EIE-2 regulates Tregs in RA and its underlying therapeutic target remain unclear. Previous studies have shown that the activation of Syk, mTOR, and PKCθ is implicated in the development and progression of RA (22, 27, 62, 78). Syk can trigger various branches, such as the mTOR (25) and PKC signaling pathways (28–32). Interestingly, this study has revealed that EIE-2 bidirectionally regulates the PKCθ/mTOR ratio in Tregs by targeting Syk in the models simulating the active and inactive phases of RA. Considering the pivotal roles of PKCθ and mTOR in the repression of Treg function and the regulation of FOXP3 expression (79–81), activation of PKCθ promotes Treg development and differentiation (80), whereas mTOR activation suppresses FOXP3 transcription (26). Consequently, alterations in the PKCθ/mTOR ratio at the transcriptional level indicate that EIE-2 restores immune balance by regulating the number of Tregs, thereby improving RA-related symptoms. Specifically, by inhibiting Syk, EIE-2 upregulated the PKCθ/mTOR ratio, which increased the number of Tregs in CIA rats and mice with carrageenan-induced paw edema. However, EIE-2 also downregulated the PKCθ/mTOR ratio by inhibiting Syk, thereby decreasing the number of Tregs in control mice. Similarly, *ex vivo* experiments with isolated spleen lymphocytes showed differential regulation of PKCθ/mTOR ratio at the transcription level with or without TNF-α induction. Furthermore, this study used Jurkat cells, a type of CD4+ T-cell commonly employed in investigating Tregs signal transduction (82–84), to simulate changes in the number of Tregs during active and inactive phases of RA *in vitro*. EIE-2 significantly increased FOXP3 expression in TNF-α-treated Jurkat cells by inhibiting the Syk/mTOR pathway (resulting in an up-regulation of the PKCθ/mTOR ratio at the transcription level), but decreased FOXP3 expression in PMA-treated Jurkat cells by inhibiting the Syk/PKCθ pathway (leading to a down-regulation of the PKCθ/mTOR ratio at the transcription level). Additionally, molecular docking analyses revealed that EIE-2 fit well with the binding site of Syk, with the docking score of EIE-2 to Syk being comparable to or even surpassing that of the Syk inhibitor (GS-9876) to Syk. Furthermore, the utilization of siRNA-Syk provided additional evidence for the bidirectional regulation of the PKCθ/mTOR ratio by EIE-2 through its targeted action on Syk.

In summary, the data in this study creatively offer a new approach on treating RA with EIE-2. It elucidates that the modulation of Tregs by EIE-2 is governed via a Syk-dependent pathway involving PKCθ or mTOR signaling branches, both in models of the active and inactive phases of experimental RA. EIE-2 can effectively control the imbalance of Tregs and inflammation levels during the active phase of RA to achieve rapid remission. Additionally, it regulates the Tregs balance during the inactive phase of RA to maintain the homeostasis in the local immune microenvironment, helping to avoid the adverse effects of excessive immunosuppression. These findings may lead to the development of treatment strategies and therapeutic targets for Treg-dependent immune tolerance disorders in individuals with RA.

However, there are still some limitations in the present study. Firstly, given the absence of reported animal models for the inactive phase of RA in the previous research, we hypothesized that the active RA could be converted to the inactive state through effective drug treatments. To prevent prior medications from biasing the efficacy assessment of EIE-2, we opted to use healthy (control) mice as a proxy for the inactive RA phase. This approach undoubtedly simplifies the complexity of RA’s inactive phase. Consequently, developing a dependable and consistent animal model for the inactive stage of RA, along with robust evaluation criteria, remains a pivotal focus for our future research endeavors. Furthermore, although our findings indicate that EIE-2 restores Tregs homeostasis by targeting Syk to modulate the PKCθ/mTOR ratio, these results are based on protein expression data from animal models and mRNA data from cellular models. We plan to further evaluate the protein expression of signaling molecules in subsequent studies using gene-knockout mice and highly purified Treg cells, aiming to provide a comprehensive and in-depth elucidation of the mechanism by which EIE-2 alleviates RA.

## 5 Conclusion

In conclusion, the present data reveal for the first time that a new 3-arylbenzofuran derivative, EIE-2, could effectively alleviate the severity of rheumatoid arthritis. The underlying mechanism may involve its targeting on Syk to upregulate the PKCθ/mTOR ratio during the active phase of RA, and to downregulate the PKCθ/mTOR ratio during the inactive phase, ultimately promoting Treg-dependent tolerance restoration.

## Data Availability

The original contributions presented in the study are included in the article/supplementary material. Further inquiries can be directed to the corresponding authors.
